# Bioinductive Collagen Augmentation in Arthroscopic Rotator Cuff Repair: 24-Month MRI and Clinical Outcomes

**DOI:** 10.3390/jcm15062435

**Published:** 2026-03-22

**Authors:** Daniele De Amicis, Aurelio Picchi, Luca Andriollo, Francesco Calafiore, Michela Saracco, Riccardo Fabiani, Andrea Fidanza, Giandomenico Logroscino, Francesco Raffelini

**Affiliations:** 1 Unit of Orthopaedics Department of Life, Health and Environmental Sciences, University of L’Aquila, 67100 L’Aquila, Italy; danieledeamicis79@hotmail.it (D.D.A.); francesco.calafiore@graduate.univaq.it (F.C.); riccardo.fabiani@graduate.univaq.it (R.F.); andrea.fidanza@univaq.it (A.F.); giandomenico.logroscino@univaq.it (G.L.); 2 Val Vibrata Hospital, 64027 Teramo, Italy; 3 Robotic Prosthetic Surgery Unit and Sports Traumatology, Fondazione Poliambulanza Istituto Ospedaliero, 25124 Brescia, Italy; andriollo.luca@gmail.com; 4 Artificial Intelligence Center, Alma Mater Europaea University, 1090 Vienna, Austria; 5 Department of Public Health, Orthopaedics and Traumatology, University of Naples “Federico II”, 80138 Naples, Italy; michelasaracco@gmail.com; 6 Istituto Fiorentino di Cura e Assistenza, 50139 Florence, Italy; f.raffelini@gmail.com

**Keywords:** rotator cuff repair, bioinductive implant, collagen membrane, full-thickness tears

## Abstract

**Background/Objectives:** Rotator cuff repair (RCR) is a common orthopedic procedure, with healing outcomes strongly influenced by patient-specific factors such as tissue quality, tear characteristics, and biological healing potential. Bioinductive collagen implants have shown great results in enhancing tendon healing and reducing retear rate. This study aimed to evaluate the clinical and imaging outcomes of RCR augmented with a xeno-derived collagen membrane over 24 months and to assess complications or implant failures. **Methods:** Patients underwent arthroscopic RCR using anchors (single or double-row) with additional xeno-derived matrix augmentation. The study included patients older than 40 years with full-thickness supraspinatus and/or infraspinatus tendon tears (DeOrio–Cofield grade 3–4) who were candidates for arthroscopic rotator cuff repair and provided informed consent. Clinical outcomes were assessed using the Constant–Murley Score (CMS), Disabilities of the Arm, Shoulder and Hand score (DASH), and Visual Analogue Score (VAS) at baseline, 3, 6, 12, and 24 months. MRI was performed preoperatively and at 24 months to assess tendon thickness. **Results:** All scores improved significantly. CMS increased from 16.3 ± 4.1 to 82.9 ± 5.8, VAS decreased from 7.8 ± 1.0 to 1.5 ± 0.8, and DASH improved from 70.3 ± 6.4 to 12.4 ± 4.5 (*p* < 0.05). Tendon thickness in the supraspinatus (T3) increased from 4.2 ± 0.9 mm to 6.8 ± 1.2 mm (*p* < 0.05). Retear rate was 7.55%, with no major complications. **Conclusions:** The bioinductive collagen implant showed notable results in improving tendon thickness, healing, and excellent clinical outcomes in RCR, without membrane-related complications. The study was designed as a prospective single-arm case series without a control group and that was the main limitation; The absence of adverse reactions in this cohort further supports the favorable safety profile of this implant in the present study population.

## 1. Introduction

Rotator cuff injuries are a prevalent orthopedic concern, and as the population continues to age, the number of affected patients is expected to increase [1]. Consequently, there will be a rise in the incidence of rotator cuff tears that require surgical intervention. In addition to the athletic population traditionally affected by rotator cuff injuries, an increasing number of middle-aged and older patients maintain high functional demands and continue participating in sports activities even after the age of 40. In these patients, rotator cuff tears frequently occur in tendons that already show signs of degeneration, often characterized by reduced tissue quality and varying degrees of fatty infiltration. This combination of high functional expectations and compromised tendon biology represents a significant challenge for surgical repair and may negatively influence healing outcomes (x). Despite significant advances in the understanding of rotator cuff injuries and biomechanically sophisticated surgical techniques, a considerable proportion of repairs still fail to achieve complete tendon healing [2]. Factors such as tear size, chronicity, patient age, and muscle atrophy play a pivotal role in determining the outcomes of surgical repair. The intrinsic degeneration of the rotator cuff tendons, characterized by structural and cellular changes such as collagen fiber disorganization and fatty infiltration, is a key factor limiting the success of surgical repair, which has prompted increasing interest in biological strategies aimed at enhancing the healing environment [3]. To address these challenges, the field of bioengineering is actively exploring innovative solutions aimed at improving tendon healing and repair [4,5]. Researchers are investigating various biological and bioinductive approaches, such as scaffolds and growth factors, to enhance tissue regeneration and support the healing process [6]. However, evidence regarding the clinical effectiveness and indications of these biological augmentations remains heterogeneous, and further studies are needed to clarify their role in clinical [7,8,9,10].

The primary goals of rotator cuff repair (RCR) are to alleviate pain, restore tendon function, and reestablish the anatomical footprint [11]. However, compromised vascularity and poor tissue quality often hinder healing, resulting in an increased retear rate [12]. Goutallier et al. in 1994 developed the Goutallier index to assess fatty infiltration in the rotator cuff muscles, a parameter that has been associated with the prognosis of retear [13]. Moreover, patients with a Goutallier index greater than 2, which indicates significant muscle degeneration, often present tendons of poor quality [14]. The Goutallier index was originally described using computed tomography (CT), while Fuchs et al. developed a specific classification to analyze fatty infiltration using magnetic resonance imaging (MRI) [15]. Even in the absence of fatty degeneration, massive tears typically exhibit poor tissue quality, leading to an elevated risk of surgical failure [16]. Long-term results have shown that anatomic healing of rotator cuff tears is associated with better outcomes, but the quality of the healed tissue remains a critical factor for success [17]. Zhao et al. reported that the postoperative retear rate following arthroscopic RCR is significantly influenced by factors such as age, stump classification, and tear size [18]. In particular, the stump classification, through its ability to reflect the fragility of the tendon, emerges as a crucial indicator for predicting the likelihood of retear [19].

These challenges have spurred interest in biologic augmentations [7]. Recent studies have revealed that graft and scaffold augmentations, particularly acellular human dermal allograft and bovine collagen, yield promising results in RCR [8]. Meta-analyses suggest that biological augmentation may reduce retear rates in selected patients, although the quality of the available evidence and the heterogeneity of the reported techniques warrant cautious interpretation [9,10]. The present study aimed to evaluate clinical outcomes, to assess the thickness of the regenerated tendon, and to determine the rate of postoperative failures after arthroscopic rotator cuff repair augmented with a bioinductive collagen scaffold. By reporting both clinical and imaging outcomes, this study seeks to contribute additional evidence regarding the potential role of bioinductive implants in rotator cuff repair.

## 2. Materials and Methods

The present study was designed as a prospective single-arm case series. Between 2020 and 2022, arthroscopic RCR augmented with a bovine bioinductive collagen implant (Regeneten^®^, Smith & Nephew, Inc., Andover, MA, USA) was performed. This study was reported in accordance with the STROBE (Strengthening the Reporting of Observational Studies in Epidemiology) guidelines for observational studies, adapted for case series.

All surgeries were performed by two surgeons exclusively, following a standardized surgical and rehabilitation protocol. Surgeries and follow-ups were conducted at Val Vibrata Hospital, Sant’Omero, Teramo, Italy, and the Istituto Fiorentino di Cura e Assistenza, Florence, Italy. Ethical approval was obtained, and informed consent was secured from all participants. Inclusion criteria were age >40 years, full-thickness supraspinatus and/or infraspinatus tendon tears (grade 3–4 according to the DeOrio–Cofield classification), Bernageau–Patte stage 3, Goutallier–Fuchs stage 3–4, indication for arthroscopic rotator cuff repair, and provision of informed consent. Exclusion criteria were partial-thickness rotator cuff tears (SCOI classification A1–A3 and B1–B3), shoulder instability, SLAP lesions requiring repair, shoulder pseudoparalysis, BMI > 35, intra-articular injections within 3 months before surgery, medical contraindications to arthroscopic surgery, and lack of informed consent. All inclusion and exclusion criteria applied in the study are outlined in Table 1.

The DeOrio–Cofield classification was used to categorize the type of rotator cuff tear pre-operatively. The study included patients with full-thickness supraspinatus and infraspinatus tendon tears.

In the present study, the biomaterial was specifically applied in a selected subgroup of patients presenting with massive rotator cuff tears, a condition characterized by high heterogeneity in tissue quality and healing potential. Rotator cuff tears were considered massive in the presence of Bernageau–Patte stage 3 tendon retraction, Goutallier–Fuchs grade 3 or 4 fatty degeneration, or a positive tangent sign. The surgical strategy was therefore personalized by adopting an arthroscopic repair with biomaterial augmentation, tailoring the intervention to patients at higher risk of failure with standard repair alone.

The baseline data collected included medical history of patients, covering conditions such as diabetes, smoking habits, and previous shoulder injuries. Key details related to the injury, such as the timing of the incident, history of trauma, symptoms duration, and prior treatments, were also documented. Exclusion criteria encompassed arthritis, age below 40 years, partial tears of the rotator cuff, shoulder instability in anterior, posterior, or multidirectional patterns, SLAP lesion at the biceps anchor, recent intra-articular injection of hyaluronic acid or corticosteroids (within 3 months prior to the planned surgery) and other severe comorbidities contraindicating elective surgical treatment.

The indication for patch augmentation was based on intraoperative assessment of tendon quality and tear characteristics, including large or massive tears and poor tendon tissue quality associated with degenerative changes such as fatty infiltration.

Clinical outcomes were assessed using the Constant–Murley score (CMS), Disabilities of the Arm, Shoulder, and Hand score (DASH), and Visual Analog Scale (VAS) at baseline, and at three, six, twelve and twenty-four months after surgery. Radiological assessments were performed using a standardized MRI technique to evaluate the rotator cuff tendons. MRI evaluations were conducted preoperatively and at twenty-four months.

Thickness was measured at baseline and at twenty-four months in three specific points along the rotator cuff tendon, designated as T1, T2, and T3, which were located at the muscle, the musculotendinous junction and in the tendinous portion. To standardize measurements and reduce interobserver variability, the anatomical landmarks used for these points were defined according to the tendon stump landmarks described in the stump classification introduced by Ishitani and colleagues [19]. Additionally, fatty infiltration was classify according to Fuchs [15]. The quality of tendon was assessed using the Stump Classification based on the C/D ratio, both preoperatively and at 24 months post-surgery [19]. MRI of the operated shoulder was performed to assess tendon integrity and retear rate, following the classification by Sugaya et al., with types IV and V indicating retears [20].

The MRI scans were analyzed independently by two blinded radiologists, ensuring an objective evaluation process. Interobserver agreement between the two blinded radiologists was assessed using Cohen’s kappa coefficient, demonstrating excellent agreement (κ = 0.87). The MRI examinations were performed using a Magnetom Sola 1.5 Tesla permanent magnet (Siemens Healthineers GmbH, Erlangen, Germany), following a protocol that included routine sequences such as axial TSE T2-weighted, coronal T1-weighted, proton density fat-sat, and sagittal thin-slice T2-weighted imaging, along with specialized sequences designed to evaluate tendons.

Bioinductive collagen implant

The Regeneten™ bioinductive implant (developed by Smith & Nephew plc, Andover, MA, USA) is an absorbable scaffold composed of highly purified type I collagen. It is designed to be placed over the site of tendon repair to provide a biological matrix intended to healing process.

According to the manufacturer, the implant promotes fibroblast proliferation and extracellular matrix formation at the repair site, potentially facilitating tendon healing. The scaffold gradually resorbs over a period of approximately six to twelve months. During this time, the device is intended to be replaced by newly formed tendon-like tissue capable of bearing functional loads, thereby avoiding the presence of permanent foreign material.

The bioinductive collagen scaffold is engineered to allow cellular infiltration and collagen deposition, features that are intended to support tendon repair procedures. However, the extent of these effects and their clinical relevance depend on surgical context.

**Surgical Technique**

**Figure 1:**
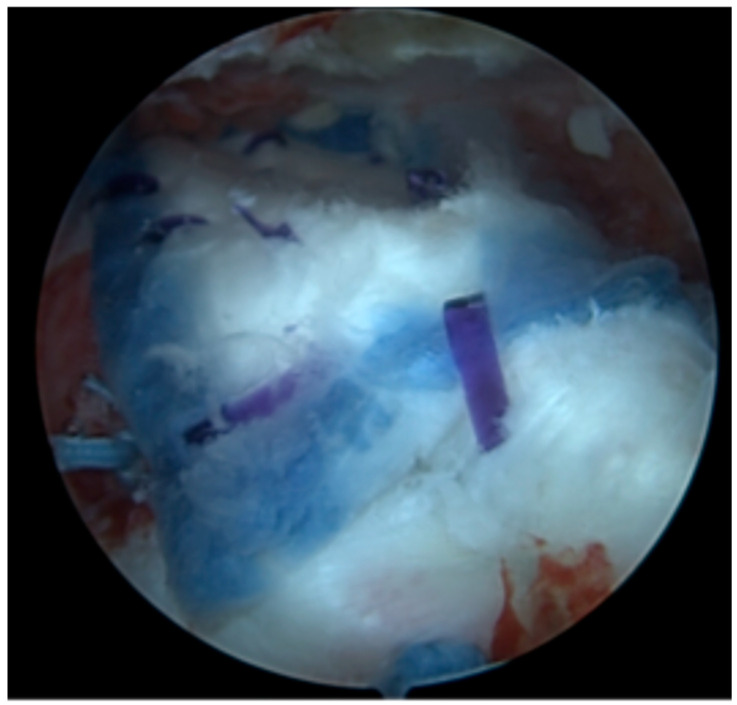
Arthroscopic final view.

All patients underwent surgery following a standardized surgical protocol performed by two experienced surgeons. The procedures were conducted under general anesthesia, with patients positioned in the “beach-chair” position to facilitate optimal access to the shoulder joint. Patients with grade III, according to the DeOrio–Cofield classification, underwent arthroscopic RCR using the single-row technique, whereas patients with grade IV tear were treated with double-row technique. For tendon repair, healicoil regensorb 5.5 mm Suture Anchors (Smith & Nephew plc, Andover, MA, USA) were used, providing secure and reliable fixation. To enhance the healing process and protect the repaired tendon, the Regeneten^R^ implant (Smith & Nephew plc, Andover, MA, USA) was applied as an augmentation scaffold over the repair site (Figure 1).

This combination aimed to improve biomechanical stability, promote tendon regeneration. The consistent use of this protocol across all patients ensured uniformity in the surgical approach, allowing for reliable assessment of clinical and imaging outcomes. Information regarding concomitant procedures has been reported. Specifically, any additional procedures performed during surgery, as well as previous surgical interventions involving the affected shoulder, have been reported.

Rehabilitation protocol

All the patients enrolled in the study followed the same rehabilitation protocol, which was applied consistently to ensure the reliability and consistency of the results. The program was developed to meet the specific postoperative needs of patients following rotator cuff reconstruction, with each phase of treatment carefully monitored to maximize functional recovery and reduce the risk of complications. To ensure standardization of the process, all patients were followed in the same center of physiotherapists, who oversaw the entire rehabilitation journey, ensuring that each patient adhered to the prescribed timelines and methods of intervention. This approach helped minimize variability in treatments and allowed for a more precise assessment of the effectiveness of the rehabilitation protocol in improving shoulder mobility and strength post-operatively. Postoperative rehabilitation included an initial phase of neutral immobilization with arm positioned at 15° of abduction for three weeks, during which only passive range-of-motion exercises were allowed and active mobilization of the elbow. Passive mobilization was progressively introduced after the immobilization period, followed by active-assisted and active movements from approximately five weeks postoperatively, while strengthening exercises were generally initiated after seven weeks. Adherence to the rehabilitation protocol was monitored by the physiotherapists at the rehabilitation center through regular follow-up sessions, and any deviations from the prescribed protocol were documented and managed according to clinical progression of patient.

Statistical Analysis

Data analysis was conducted using Jamovi software (v 2.3; The Jamovi Project, Sydney, Australia). Continuous variables were summarized as mean ± standard deviation (SD), and categorical variables were expressed as frequencies and percentages. A post hoc power analysis was performed considering the Constant Score as the primary outcome measure, with an alpha level of 0.05. Based on the observed variability in the dataset, the available sample of fifty-three patients provided a statistical power greater than 0.90 to detect clinically relevant differences over time.

Changes in outcomes across multiple follow-up time points were primarily assessed using repeated-measures analysis of variance (ANOVA). Statistical significance was determined at a threshold of *p* < 0.05, with 95% confidence intervals reported for all comparisons. Normality of the data was evaluated using the Shapiro–Wilk test.

## 3. Results

Fifty-three consecutive patients (twenty-two females, thirty-one males) were followed, with a mean age of 62.81 ± 9.3 years. The patients were distributed according to the DeOrio–Cofield classification as follows: 15 patients were categorized as grade III, 38 as grade IV. Significant improvements were observed across all clinical outcomes from baseline to the 24-month follow-up. CMS improved progressively, starting from a preoperative mean of 16.3 ± 4.1 and increasing to 45.2 ± 8.1 (*p* < 0.05) at three months, 55.6 ± 7.3 (*p* < 0.05) at six months, 70.1 ± 6.9 (*p* < 0.05) at twelve months, and finally reaching 82.9 ± 5.8 (*p* < 0.05) at twenty-four months. The overall improvement from baseline to 24 months was 66.6 points (95% CI 63.4–69.8). DASH demonstrated a consistent reduction, decreasing from 70.3 ± 6.4 preoperatively to 58.5 ± 6.9 (*p* < 0.05) at three months, 40.7 ± 8.1 (*p* < 0.05) at six months, 28.5 ± 7.4 (*p* < 0.05) at twelve months, and reaching 12.4 ± 4.5 (*p* < 0.05) at twenty-four months. The mean reduction from baseline to 24 months was 57.9 points (95% CI 54.8–61.0). VAS for pain also showed significant improvement, decreasing from a preoperative mean of 7.8 ± 1.0 to 4.8 ± 1.2 (*p* < 0.05) at three months, 1.4 ± 1.4 (*p* < 0.05) at six months, 1.7 ± 1.2 (*p* < 0.05) at twelve months and 1.5 ± 0.8 (*p* < 0.05) at twenty-four months. The mean decrease from baseline to 24 months was 6.3 points (95% CI 5.9–6.7). All clinical outcomes are reported in Table 2.

**Figure 2:**
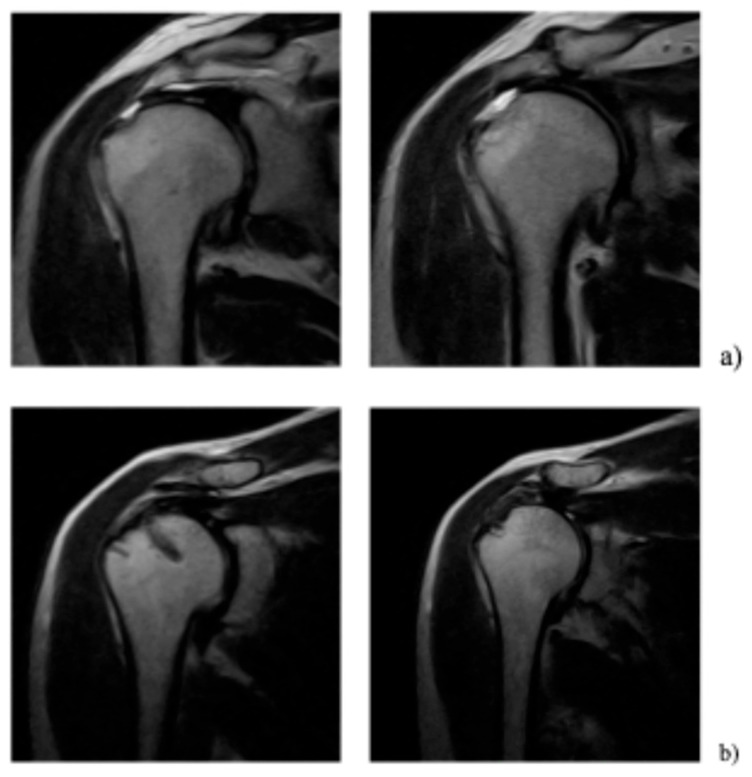
Magnetic resonance before surgery (**a**); magnetic resonance at 1 year of follow-up (**b**).

The thickness of the tendon in the most lateral area (T3), located in the tendinous portion of the supraspinatus tendon, significantly increased during the follow-up (Figure 2).

Specifically, it changed from a mean value of 4.2 mm ± 0.9 mm preoperatively to 6.8 mm ± 1.2 mm at 24 months postoperatively, with a statistically significant difference (*p* < 0.05). No significant changes were observed in the more medial zones, T1 and T2 (Table 3).

The thickness in the T1 zone (muscular Region) remained relatively stable, measuring 12.8 mm ± 1.5 mm preoperatively and 13.1 mm ± 1.4 mm at 24 months (*p* = 0.321). Similarly, in the T2 zone (musculotendinous junction), the thickness changed minimally, from 7.4 mm ± 1.2 mm preoperatively to 7.6 mm ± 1.1 mm at 24 months (*p* = 0.542). At 24 months postoperatively, patients with intact repairs were consistently classified as Sugaya Grade II on MRI evaluation. The overall complication rate throughout the study was low, with a retear rate of 7.55% (2/53 patients). Both retears occurred in patients with large tears (Cofield grade IV) and were associated with early anchor dislocation, which led to mechanical failure of the repair and required additional surgical intervention. On MRI evaluation, these two cases were classified as Sugaya Grade V, consistent with a full thickness retear. These failures were therefore considered mechanical rather. No other serious adverse events related to the surgical procedure or the implant were encountered. Furthermore, no adverse events directly linked to the augmentation procedure were documented.

## 4. Discussion

In arthroscopic RCR, the quality of repaired tendon is a crucial factor for clinical success and reduction in postoperative complications [21,22]. Previous studies have shown that postoperative complications and failure of tendon healing are significantly more frequent in patients with compromised tendon quality compared to those with a torn tendon that remains structurally intact and free of degeneration or fraying [23,24,25]. In patients with severely deteriorated tendon quality, healing is considerably lower, highlighting the importance of a thorough preoperative assessment of tendon tissue [26].

Subcutaneous patches derived from porcine small intestine have been explored for RCR but concerns remain regarding their biocompatibility. In particular, the study by Iannotti et al. reported severe tissue reactions and rejection associated with a porcine-derived patch [27]. Similarly, other xenogeneic patches have shown adverse tissue responses, raising questions about their safety and long-term integration [28]. Several studies, including the present one, have not reported any foreign body reactions or signs of tissue response, either clinically, through ultrasound (US), or via MRI, indicating a favorable safety profile [29,30,31]. In both animal models and human trials with arthroscopic second-looks, xeno-derived collagen implants were fully integrated into native tissue within 6 months [8,32].

In addition, other mid-term studies on bovine-derived patches have similarly found no adverse reactions to the graft, reinforcing the safety and viability of these materials for clinical use [7,33].

The case report published by Serra et al. describes the only one documented instance of Regeneten^®^ implant migration with acromial lodging in asymptomatic pattern, highlighting the importance of MRI follow-up [34].

The results of this study revealed promising outcomes in terms of both tendon healing and safety. Notably, at 24 months, no foreign body reactions, tissue rejection, infections or adverse events related to the implant were observed, which is consistent with earlier studies using this bioinductive scaffold. This is an important finding, as xenograft products have raised safety concerns in the past, particularly regarding immune responses. The absence of adverse reactions in this cohort further supports the favorable safety profile of this implant in the present study population.

Warren et al., in a meta-analysis, analyzed the outcomes of rotator cuff repairs augmented with bioinductive collagen patches, emphasizing their effectiveness in enhancing tendon healing and reducing retear rates compared to conventional techniques [35]. The primary outcomes assessed included the American Shoulder and Elbow Surgeon (ASES) score, CMS, pain levels quantified using VAS, changes in tendon thickness, and the incidence of complications. Bryant et al. found no evidence that xenogeneic-derived patch provides superior outcomes in patients with moderate rotator cuff tears [36]. On the contrary Consigliere et al. found excellent clinical outcomes in their sample; these findings suggest that, in addition to the structural integrity outcomes, the use of extracellular porcine dermal matrix augmentation contributed to meaningful functional recovery and shoulder function restoration in patients with medium to massive rotator cuff tears [37].

A common complication after RCR is tendon retear, with failure rates ranging from approximately 7% to 17% for small to medium tears, 41% to 69% for large tears, and 40% to 94% for massive tears [38]. The retear rate in patients undergoing RCR without augmentation, but with an intact subscapularis tendon, has been reported to be around 20% [39]. Our sample showed an overall retear rate of 7.55%, which is consistent with findings from other authors who also reported lower retear rates in patients treated with the Regeneten implant compared to those undergoing standard repair without bioinductive augmentation [40,41,42]. While the observed retear rate compares favorably with previously published data, the absence of a contemporaneous control group should be acknowledged, as the outcomes observed in this cohort may also reflect surgical expertise, standardized rehabilitation, and careful patient selection rather than the implant effect alone.

One of the most significant findings from this study is the observed increase in tendon thickness from 4.2 mm preoperatively to 6.8 mm in T3 zone at 24 months, as measured by MRI. This increase in tendon thickness is in line with previous studies that documented the ability of bioinductive collagen scaffolds to stimulate tendon remodeling and healing [3,43,44,45].

Yeazell et al. reported a significantly higher rate of postoperative frozen shoulder in patients treated with a bioinductive collagen patch for high-grade partial-thickness rotator cuff tears [46]. Notably, all cases of stiffness requiring reoperation occurred in the patch group, highlighting a potential complication associated with this treatment approach. In a recent systematic review regarding the use of collagen implants in RCR, similarly high rates of reoperation for stiffness, as reported by Yeazell et al., were not observed [47]. While some studies did note the occurrence of postoperative stiffness, the incidence was generally low and did not require surgical intervention. In our study, no patients experienced shoulder stiffness or adhesive capsulitis after surgery. This is consistent with most studies in the literature, where stiffness was rare and usually did not need further treatment [41,42,45,48,49]. The absence of postoperative stiffness requiring intervention in our cohort may be related to factors such as standardized rehabilitation protocols, careful patient selection, and surgical technique, rather than reflecting an intrinsically lower risk associated with the implant itself.

Only two randomized controlled trials exist evaluating bioinductive collagen implants in rotator cuff repair [41,50]. Both studies demonstrated improved tendon healing and lower retear rates compared to standard repairs. Notably, neither study reported an increased rate of complications, supporting the virtuous profile of the augment.

The scaffold promotes a favorable biological environment for tendon regeneration, including enhanced vascularity and collagen formation [51,52,53]. While the current study provides valuable insights into the potential benefits of using a bioinductive collagen scaffold in rotator cuff repair, there are several limitations that should be considered. First, the absence of a control group makes it difficult to draw direct comparisons between the Regeneten^R^-augmented repairs and traditional repair techniques. During the study period, augmentation was considered for patients presenting these high-risk features; however, the final decision was made according to the surgeon’s clinical judgment, which may have introduced a degree of selection bias. Additionally, the study was not randomized, which may introduce selection bias. Future studies with randomized controlled designs and longer follow-up periods are needed to confirm the long-term benefits of this bioinductive implant and establish its role in clinical practice. Another limitation of the present study is the absence of objective strength testing or functional performance measures. Clinical outcomes were evaluated primarily using patient-reported outcome measures, which, although widely validated, may not fully capture objective changes in muscle strength or functional shoulder performance following rotator cuff repair.

Furthermore, while the 2-year follow-up in this study was sufficient to evaluate tendon healing and retear rates, longer follow-up is necessary to assess the long-term durability of the implant and its impact on clinical outcomes such as strength and functional recovery.

## 5. Conclusions

The results of this study suggest that the bioinductive collagen scaffold represents a promising adjunct in rotator cuff repair surgery, particularly for selected patients with compromised tendon quality and an increased risk of retear. In this prospective case series, the implant was associated with increased tendon thickness and low retear rates, while also demonstrating a favorable safety profile. Within a personalized treatment strategy, the use of a bioinductive collagen scaffold may therefore represent a valuable option for patients at higher risk of healing failure. However, these findings should be interpreted within the limitations of the present study design. Future randomized controlled trials directly comparing augmented and non-augmented repairs in clearly defined high-risk patient groups are warranted to further clarify the clinical benefits of this approach.

## Figures and Tables

**Table 1:** Inclusion and exclusion criteria.

Inclusion Criteria
Age >40 years old
Full-thickness supraspinatus and/or infraspinatus tendon tears (grade 3 and 4 according to DeOrio–Cofieldclassification).
Bernageau-Patte stage 3
Goutallier-Fuchs Stage 3 and 4
Indication for arthroscopic rotator cuff repair.
Informed consent to participate in the study.
**Exclusion criteria**
Partial tears of the rotator cuff tendons (categorized as A1, A2, A3, B1, B2, and B3 in the SCOI classification).
Shoulder instability in anterior, posterior, or multidirectional patterns.
Cases requiring repair of a SLAP lesion at the biceps anchor.
Pseudo paralysis of the shoulder.
Recent intra-articular injection of hyaluronic acid or corticosteroids (within 3 months prior to the planned surgery).
Medical conditions that contraindicate arthroscopic shoulder surgery.
Cases where informed consent for the procedure is not granted

**Table 2:** All clinical results.

	Pre-Operative	At 3, 6, 12 and 24 Months	*p*-Value
**Constant–Murley score**	16.3 ± 4.1	45.2 ± 8.1 55.6 ± 7.3	<0.05 <0.05
		70.1 ± 6.9 82.9 ± 5.8	<0.05 <0.05
**DASH**	70.3 ± 6.4	58.5 ± 6.9 40.7 ± 8.1 28.5 ± 7.4 12.4 ± 4.5	<0.05 <0.05 <0.05 <0.05
**VAS**	7.8 ± 1.0	4.8 ± 1.2 1.4 ± 1.4 1.7 ± 1.2	<0.05 <0.05 <0.05
		1.5 ± 0.8	<0.05

**Table 3:** Tendon thickness in the muscular region (T1), musculotendinous junction (T2), tendon region (T3).

	T1 (mm)	T2 (mm)	T3 (mm)
Pre-operative MRI	12.8 ± 1.5	7.4 ± 1.2	4.2 ± 0.9
Control-MRI (twenty-four months)	13.1 ± 1.4	7.6 ± 1.1	6.8 ± 1.2
*p*-value	*p* = 0.321	*p* = 0.542	*p* < 0.05

## Data Availability

The data presented in this study are available on request from the corresponding authors.
